# Introduction to Topical Collection: Changing Values and Energy Systems

**DOI:** 10.1007/s11948-024-00497-x

**Published:** 2024-08-09

**Authors:** Joost Alleblas, Anna Melnyk, Ibo van de Poel

**Affiliations:** Department Values, Technology & Innovation, TU Delft, Jaffalaan 5, Delft, 2628 BX The Netherlands

**Keywords:** Value, Vale change, Energy, Energy system, Energy transition, Ethics, Design

## Abstract

This paper is the introduction to a topical collection on “Changing Values and Energy Systems” that consists of six contributions that examine instances of value change regarding the design, use and operation of energy systems. This introduction discusses the need to consider values in the energy transition. It examines conceptions of value and value change and how values can be addressed in the design of energy systems. Value change in the context of energy and energy systems is a topic that has recently gained traction. Current, and past, energy transitions often focus on a limited range of values, such as sustainability, while leaving other salient values, such as energy democracy, or energy justice, out of the picture. Furthermore, these values become entrenched in the design of these systems: it is hard for stakeholders to address new concerns and values in the use and operation of these systems, leading to further costly transitions and systems’ overhaul. To remedy this issue, value change in the context of energy systems needs to be better understood. We also need to think about further requirements for the governance, institutional and engineering design of energy systems to accommodate future value change. Openness, transparency, adaptiveness, flexibility and modularity emerge as new requirements within the current energy transition that need further exploration and scrutiny.

## Introduction

The extraction and exploitation of energy sources is - and has always been - vital for human survival. In the past - and still in some parts of the world - people used wood, water, and wind energy in daily activities, like cooking, heating, and domestic goods production. Many focal practices have developed around energy production, consumption, and supply. One might think of wood-gathering for the stove or storytelling around the campfire. As energy sources changed, social practices changed as well. Since the Industrial Revolution, energy needs have dramatically increased, facilitating urbanization and economic growth (Mitchell, 2009). In most parts of the world, fossil fuels became the energy source that determined how systems for producing, distributing, and supplying energy were designed, built, and regulated (Jamieson, 2011). Electricity, natural gas, and oil made new ways of living possible.

However, later in the twentieth century, fossil fuel-based energy systems came to be seen as a critical contributor to greenhouse gas (GHG) emissions to the atmosphere and have been under the spotlight in climate change mitigation (Singer, 2010). For over a decade, numerous policy proceedings, reports and directives have indicated the need to transition to renewable energy sources (e.g., Edenhofer et al., 2011; European Union, 2018; IPCC, 2023). Nevertheless, changing the energy sector to address these concerns is a multifaceted and complex task that also requires an inquiry into underlying values.

Values play an important role in navigating the complexity of the energy transition and in the (re)design of energy systems (e.g., Künneke et al., 2015; Oosterlaken, 2015; Van de Poel, 2017; Taebi & Kloosterman, 2015; Mok & Hyysalo, 2018; Milchram et al., 2019; Melnyk et al., 2023). At the same time, values are dynamic and may change after a system has been designed (Van de Poel, 2021). Therefore, a mismatch may occur between the values embedded in the past when designing an energy system and the new and emerging values people find essential today. For example, sustainability and energy autarky are new values discussed in public, legal, and academic debates around the energy transition (e.g., Demski et al., 2015; Van de Poel & Taebi, 2022). With some success, engineers and policymakers are trying to embed these and other values in technological and institutional designs to meet new societal expectations and contribute to the Sustainable Development Goals (SDG).

Besides a change in technologies and energy sources, embedding these and other values would also mean a change in values underlying institutions (e.g., Milchram et al., 2019; Melnyk et al., 2023). For instance, energy democracy is newly emerging value that challenges existing energy systems and institutions by bringing to the table concerns about sustainability, democracy, recognition, empowerment, and social justice (Fairchild & Weinrub, 2017) as well as other, less explored values, like community (Szulecki & Overland, 2020) and diversity (Melnyk et al., 2023). These values require a different institutional arrangement that is not limited to the availability of technological choices on the market and altering the role of citizens from “consumers” to “prosumers” (i.e., citizens as producers of energy).

Thus, although value change poses numerous design and governance challenges, it still needs further exploration. This topical collection contains interdisciplinary contributions that clarify the role of changing values in the design and governance of existing and emerging energy systems.

We start this editorial with a brief overview of conceptions of value and value change used by contributions to this topical collection, followed by a more detailed elaboration on the role of values in the design of energy systems. Then we will introduce the six contributions to this topical collection.

## Conceptions of Value and Value Change

Values express what is ‘good’ and ‘desirable.’ However, this general characterization of value still leaves open several more specific conceptualizations of the notion. An often-used conceptualization of value in the social sciences is that proposed by Schwartz and Bilsky (1987), who define values as “ (a) concepts or beliefs, (b) about desirable end states or behaviors, (c) that transcend specific situations, (d) guide selection or evaluation of behavior and events, and (e) are ordered by relative importance.” Goyal and Iychettira (2022) use this notion in their contribution to this topical collection, although they see values not just as located in someone’s personality but also as socially shared anchors or resources that express what is good.

Several papers in this topical collection – like those by Kozlovski (2022), by Timmermann and Noboa (2022), and by Frigo et al. (2023), focus on what may be called *moral values*. Frigo et al. (2023) conceptualize care not just as a moral value but also as a guiding moral mid-level principle and scrutinize caring as a process. Kozlovski (2022) is concerned with how we can deal with conflicting values in Value Sensitive Design (VSD) of energy systems and therefore focuses on the possible incommensurability of values. Timmermann and Noboa (2022) discuss energy sovereignty as a new moral value that is composed of a range of other moral values.

Correljé et al. (2022) offer a new conceptualization of values. They believe that such a new conceptualization is needed to find a middle way between more sociological notions of value that, in their view, put too much stress on the contingency of values and the way moral philosophers have traditionally understood (moral) values, which tends to see them as eternal and unchangeable. Inspired by pragmatist philosophy (Dewey) and Original Institutional Economics (OIE), they propose an understanding in which values are socially situated but also express what is (believed to be) morally desirable. One of the attractive features of their proposal is that it allows talking about value change while keeping the moral or normative connotations that come with moral values.

Alleblas (2024), in his contribution, draws a distinction between values and ideals. Ideals may be seen as idealized values that have acquired properties such as being uncompromising, not fully realizable, and navigational. Consequently, he argues that whereas values may change over time, ideals are more likely to persist.

This brings us to the theme of value change, which is addressed by several of the authorsof this topical collection. Goyal and Iychettira (2022) are, like Alleblas (2024), mainly interested in the social dynamics of value change. They propose a multiple streams framework in relation to energy policy, based on the work of Kingdon (1984) and others, in which the pace and type of value change may be different in the various streams (problem framing, policy design, political decision-making, technological diffusion).

We already mentioned how Correlje et al. (2022) develop a notion of value that allows for value change; more generally, they hold that OIE provides a useful framework to study the role of values and value change in the energy transition. The other contributions do not very explicitly address the theme of ‘value change’, but they nevertheless touch on it in various ways. Timmermann and Noboa‘s (2022) discussion of ‘energy sovereignty’ may be interpreted in terms of the emergence of a new value. Frigo et al. (2023) discuss how value change can be addressed in the design process through the value of care.

### Values and Design

One way of dealing with the question of how to let technologies and technological systems contribute to flourishing individual and societal life is through Value Sensitive Design (VSD). This approach intends to ‘frontload’ (Van den Hoven et al., 2015) ethical concerns in the early stages of design processes instead of having these concerns emerge after introducing a new technology or changing a system. These ethical concerns are addressed in the forms of values, norms, and design requirements (Van de Poel, 2013); the latter allows these technologies to embed these values, making it easier for individuals, communities, and societies to realize them.

This approach investigates what people find important in life as goals (Friedman & Hendry, 2019) and then proceeds to investigate how these goals can become part of the design of an artifact or system. This includes institutional design as well. Values such as privacy, autonomy, safety, and universal access are conceptualized, operationalized into norms, and then further translated into design requirements. This process is iterative and can proceed both ways (from existing norms and design specifications to values and from abstract values to concrete requirements). The literature onVSD further distinguishes between value conflicts (and resulting trade-offs), value thresholds, and value dams (Davis & Nathan, 2015; Van de Poel, 2015). This first occurs when augmenting the possible realization of one goal (e.g., safety) decreases the possible realization of another goal (e.g., autonomy). Value thresholds, such as safety protocols and regulations, are minimum requirements for a certain embedded value. Value dams, finally, occur when a (small) group of stakeholders is vehemently opposed to a certain design feature, such as cameras in trains (because of strong privacy concerns). In the above terminology, innovation is perceived as eliminating value conflicts, raising value thresholds, and taking away value dams.

TheVSD approach thus allows for both innovation and design to be perceived as important ethical practices, always involving questions about the good life, human flourishing, and issues such as responsibility. However, this approach can become more complicated once we design for improvements in sociotechnical systems with a multigenerational lifespan (Davis & Nathan, 2015). As thistopical collection shows, embedding certain values in an energy system can lead to temporal value conflicts since we cannot know what kind of goals future generations will hold. Certain design decisions that seem reasonable in the current situation might become problematic in the future. Innovation, therefore, has a fourth mandate: to make sure that our current designs allow for realizing goals that future generations might come to hold. This requirement would introducefurther design directives: openness, transparency, adaptiveness, flexibility, modularity, and so forth (Van der Weij et al., 2023).

Several contributions in this topical collection highlight how this relation to the future plays out in the context of designing for values. Alleblas (2024) shows, inter alia, how certain idealized values came to dominate political discussions of a tidal power project in the UK, which both stalled the realization of the project and prevented it from being abandoned. Timmerman and Noboa (2022) show how an emerging value, energy sovereignty, can be conceptualized using an existing value, food sovereignty. They emphasize how these conceptual investigations help to define normative principles for energy sovereignty that are much needed to bridge the divide between diverse stakeholders in an energy sector in transition. Frigo et al. (2023) discuss a complementary approach to VSD: Designing for Care (D4C). D4C, they argue, can have multiple positive effects and may offer moral guidance to overcome difficulties arising in three types of value change.

### About the Contributions

#### Giovanni Frigo, Christine Milchram, and Rafaela Hillerbrand

discuss a new and original approach that offers moral guidance to overcome difficulties arising from some types of value change. Designing for Care (D4C) is a distinctive approach to technological design and responsible innovation informed by care ethics and a relational ontology of situated agents.A case study of a community battery project shows how D4C would affect the design process of such projects and, moreover, how D4C would impact the practitioners involved in achieving the energy transition.

#### Cristian Zimmerman and Eduardo Noboa

investigate the concept of energy sovereignty, comparing it to food sovereignty and emphasizing conceptual overlaps. This comparison leads to a set of normative principles for energy sovereignty that helps coordinate the actions of a diverse set of stakeholders in the energy transition, emphasizing shared concerns rather than differences. Zimmerman and Noboa emphasize the role of conceptual development and community in the ongoing energy transition. They imply that energy sovereignty is fulfilling both roles.

#### Joost Alleblas

focuses on how ideals have come to dominate certain discussions of energy system innovation. With his analysis, Alleblas shows how ideals can both motivate and coordinate change but also lead to stagnation, especially as ideals and idealized technologies become intertwined in what he calls ‘utopian frames’. Alleblas further emphasizes that the co-evolution of values and technologies, characteristic of sociotechnical systems, does not necessarily have to lead to new visions as collectively held ideas of desirable futures that instigate sociotechnical change.

#### Atay Kozlovski

discusses conflicts between values in Value Sensitive Design. Building on ideas from the philosopher Ruth Chang (2022), he holds that sometimes such conflicting values may be ‘on a par’ so that there are no value-based reasons to prioritize one value over the other. Rather such cases, he argues, can be rationally resolved through volitational commitments, which can be individual as well as collective. He explores which actors are entitled to make such decisions and when decision-making is political rather than ethical.

#### Nihit Goyal and Kaveri Iychettira

discuss values in policy processes in energy transitions. They argue that, while policy innovation is essential for upscaling responsible innovation, it also leads to contestation and value conflicts. The authors use the multiple streams framework (MSF) to show that the values that ‘govern’ problem framing, policy design, political decision making, and technological diffusion can evolve relatively independently, potentially leading to value conflict. After applying this framework to the market-based economic dispatch of electricity (MBED) policy in the Indian energy transition, the authors conclude that a disaggregation of values, based on the MSF, can facilitate an analysis of value change and value conflict in sociotechnical transitions.

#### Aad Correljé, Udo Pesch, and Eefje Cuppen’s

contribution signifies the role of values in institutional change within the ongoing energy transition by scrutinizing the interplay between technologies, institutions, and value change. The authors’ perspective on values is grounded on Dewey’s pragmatist account and explicates that values shape individual choices and impact social order. Building on the insights from Original Institutional Economics and Dewey’s account of values, this contribution develops a theoretical framework that aids the understanding of how changes in sociotechnical systems affect normative evaluation and values and vice versa.

## Data Availability

Not applicable.
